# Retrospective Study on the Seasonal Forecast-Based Disease Intervention of the Wheat Blast Outbreaks in Bangladesh

**DOI:** 10.3389/fpls.2020.570381

**Published:** 2020-11-23

**Authors:** Kwang-Hyung Kim, Eu Ddeum Choi

**Affiliations:** ^1^Climate Prediction Department, APEC Climate Center, Busan, South Korea; ^2^Pear Research Institute, National Institute of Horticultural & Herbal Science, Naju, South Korea

**Keywords:** disease epidemiological model, seasonal disease risk, early warning system, climate reanalysis, global crop calendar, winter wheat

## Abstract

Seasonal disease risk prediction using disease epidemiological models and seasonal forecasts has been actively sought over the last decades, as it has been believed to be a key component in the disease early warning system for the pre-season planning of local or national level disease control. We conducted a retrospective study using the wheat blast outbreaks in Bangladesh, which occurred for the first time in Asia in 2016, to study a what-if scenario that if there was seasonal disease risk prediction at that time, the epidemics could be prevented or reduced through prediction-based interventions. Two factors govern the answer: the seasonal disease risk prediction is accurate enough to use, and there are effective and realistic control measures to be used upon the prediction. In this study, we focused on the former. To simulate the wheat blast risk and wheat yield in the target region, a high-resolution climate reanalysis product and spatiotemporally downscaled seasonal climate forecasts from eight global climate models were used as inputs for both models. The calibrated wheat blast model successfully simulated the spatial pattern of disease epidemics during the 2014–2018 seasons and was subsequently used to generate seasonal wheat blast risk prediction before each winter season starts. The predictability of the resulting predictions was evaluated against observation-based model simulations. The potential value of utilizing the seasonal wheat blast risk prediction was examined by comparing actual yields resulting from the risk-averse (proactive) and risk-disregarding (conservative) decisions. Overall, our results from this retrospective study showed the feasibility of seasonal forecast-based early warning system for the pre-season strategic interventions of forecasted wheat blast in Bangladesh.

## Introduction

Wheat blast, caused by *Magnaporthe oryzae* Triticum pathotype (MoT) (anamorph *Pyricularia oryzae*), is one of the most devastating wheat diseases with near complete yield loss (Couch and Kohn, 2002; Mottaleb et al., 2019b). Since the disease was first identified in Brazil in 1985 (Igarashi et al., 1986), it was known to present only in South America. In February 2016, however, wheat blast was detected for the first time in Asia with reports of a severe outbreak in Bangladesh. The outbreak was particularly worrisome because wheat blast could spread further to major wheat-producing areas in neighboring South and East Asian countries (e.g., India, China), thus threatening food security across the region. As a result, the wheat blast affected nearly 15,000 ha (3.5% of the total 0.43 million ha of wheat area in Bangladesh) in eight southwestern districts, *viz.*, Pabna, Kushtia, Meherpur, Chuadanga, Jhenaidah, Jessore, Barisal, and Bhola (Fig. 1 of Islam et al., 2016) with an average yield loss of 24.5% in the affected fields, equivalent to USD 1.6 million when valuing wheat at USD 149/ton (Mottaleb et al., 2018).

The most promising and long-term strategy for the mitigation of wheat blast is the development of resistant varieties against wheat blast. Although there is no proven wheat blast-resistant commercial variety, however, promising results have been achieved by many researchers during resistance assessment of wheat genotypes/lines against the blast fungus, such as BARI Gom 33 (Islam et al., 2019; Mottaleb et al., 2019a). Because development of a resistant wheat variety through conventional breeding program takes a long time, the application of chemical fungicide seems to be the most feasible, cost-effective way to apply in a short-term manner. At the moment, seed treatment to eliminate the seed-borne infection or application on the spikes using fungicides combining triazoles with strobilurins has been suggested to control the disease (Kohli et al., 2011).

Weather conditions are critical factors for the development of wheat blast disease (Farman et al., 2017). There are several wheat blast epidemiological models that correlate the weather condition to wheat blast epidemics to come up with the potential epidemiological risk of wheat blast (Cardoso et al., 2008; Fernandes et al., 2017). The models use climate data, such as temperature, relative humidity, rainfall, and solar radiation, and calculate risk values contributing to the development of wheat blast [inoculum potential (IP), spore cloud (SPOR), and infection). Such prediction based on climate data can be an efficient way to determine times and locations for surveillance and monitoring and to estimate the magnitude of climate-driven disease pressure for the season.

Recent temperature increases in Bangladesh due to global warming under climate change, especially during the winter season, indicate its potential positive impacts on the occurrence and development of wheat blast disease (Hossain and da Silva, 2012). Average winter temperatures in Bangladesh have gradually increased over the last decades (Miah et al., 2014). Since this fungal pathogen favors a temperature ranging from 20 to 30°C, the occurrence of wheat blast was expected to increase, leading to increased yield loss of wheat (Cardoso et al., 2008). In South America, severe epidemics occurred primarily in humid and warmer regions, such as Bolivia, Paraguay, and north-eastern Argentina (Kohli et al., 2011). Unusual humid and warm weather in Brazil also triggered the outbreak of wheat blast disease (Farman et al., 2017). Analysis of weather data collected from the Bangladesh Meteorological Department showed that the minimum temperature at night in 2016 increased by 1.8–6.5°C compared with 2011–2015 (Islam et al., 2019). Such warming up coupled with frequent rainfall (up to 35 mm) in February likely contributed to the outbreak of the epidemics in the wheat blast affected districts.

Seasonal disease risk prediction using disease epidemiological models and seasonal climate forecasts (SCFs) has been actively sought over the last decades, as it has been believed to be a key component in the disease early warning system for the pre-season planning of local or national level disease control. In fact, dynamic global circulation models (GCMs) have become mainstream tools to deliver SCFs, due to their promising predictive skills (Syktus et al., 2003; Power et al., 2007). Unlike weather forecasts, SCFs can be predicted with a longer lead time for an upcoming season (i.e., 3–6 months). A longer forecast lead time enables mid- to long-term decisions in agricultural farming, such as planting date selection, management of water resources and infrastructure, determination of labor recruitment, selection of crops and varieties, and determination of optimal cultivation areas and cropping systems. However, to influence the operational decision-making in a practical manner, it is important that forecasts be appropriately “contextualized” before they can positively influence decision-making (Meinke and Stone, 2005). This is because decision makers in agriculture are interested in the consequences of seasonal climate variability, rather than in the climate variability itself that the SCF implicates. One such way to contextualize forecasts is to link them with impact models, e.g., agriculturally relevant information (such as crop yield or crop loss), to strengthen the potential impacts of the forecasts when presenting them to decision makers.

Here, we conducted a retrospective analysis using the wheat blast outbreaks in Bangladesh, which occurred for the first time in Asia in 2016, to study what-if scenarios that if there was seasonal disease risk prediction at that time, the epidemics could be prevented or reduced through prediction-based interventions, and what will be the potential benefits out of adopting the SCF-based early warning service. To prove this objective, we used a wheat blast epidemiological model (Fernandes et al., 2017) and a simple wheat growth model (Zhao et al., 2019) to simulate potential wheat blast disease risk and wheat yield, separately. SCFs were bias-corrected and downscaled to hourly and daily scale weather data to be used as input variables for both models. The predictability and potential value of the resulting early warning information were evaluated against observation-based model simulations, respectively.

## Materials and Methods

### Wheat Blast Epidemiological Model and Wheat Growth Model

We adopted a wheat blast model, developed for the wheat blast epidemics in South America (Fernandes et al., 2017), to simulate the wheat blast epidemics in Bangladesh. Based on Fernandes et al. (2017), the model takes into account three major epidemic processes: survival, dispersal, and infection. Briefly, IP is defined by potential propagation of fungal inoculum on alternative hosts prior or early in the season under favorable weather conditions. As the fungal inoculum has limited lifespan during air dispersal, a SPOR density declines rapidly over time. Around crop heading stage, survived fungal inoculum in a SPOR cloud infect wheat spikes when weather conditions become conducive. Detailed infection algorithms of the wheat blast model related to weather input variables can be referred to the study by Fernandes et al. (2017).

In this study, the potential disease risk for a season was represented by the accumulated successful daily infection (INF), conditioned to an IP >30, and a SPOR >0.4 during a day favoring infection (DFI), which are simulated by the wheat blast model. Since the wheat blast model was developed and evaluated for the South American region only, we calibrated the parameters of the wheat blast model before applying it to Bangladesh. Calibration of the wheat blast model was conducted based on the disease intensity reported in each district during the 2015–2016 wheat blast outbreaks (Islam et al., 2016): 0.2% in Pabna, 2% in Kushtia, 70% in Meherpur, 44% in Chuadanga, 8% in Jhenaidah, 37% in Jessore, 1% in Barisal, and 5% in Bhola. As input weather data to the wheat blast model, we used the ERA5-Land hourly data as a gridded (0.1° × 0.1°, native resolution is 9 km) weather observation proxy (Copernicus Climate Change Service [C3S], 2019). Model parameters were adjusted by minimizing root-mean square error (RMSE) values between the normalized simulated disease risk scores and the normalized observed disease intensities of the 2015–2016 seasons for all eight districts. In this way, the calibration also reduced the potential errors originated from the systematic bias of the ERA5-Land reanalysis products compared with the ground truth weather data of the target area (Kawohl, 2020). The parameters of the calibrated wheat blast model are shown in Supplementary Table 1.

To simulate the potential wheat yield level in the study area, we adopted a simple generic crop growth model called SIMPLE (Zhao et al., 2019). The SIMPLE model includes basic physiological response functions to temperature, heat stress, drought stress, and atmospheric CO_2_ concentrations to simulate biomass and yield that are similar to observations. In fact, the SIMPLE model was already parameterized and evaluated for wheat using observations for biomass growth, solar radiation interception, and yield from field experiments data in the United States and New Zealand (Zhao et al., 2019). Zhao et al. (2019) showed that the RMSE for wheat yield using test data was 17.8% with the SIMPLE model compared with 11–24% RMSE across several wheat models (Asseng et al., 2015). However, because of its very simplistic modeling approach, the SIMPLE model has a number of limitations including the lack of response to vernalization and photoperiod effect on phenology and the lack of soil–crop nutrient dynamics. While the SIMPLE model has clear limitations due to its simplicity, there is also an advantage from its simplicity, such as its applicability to national or regional level studies with limited ground truth data. Another scaling issue from using extremely low-resolution SCFs (1–2.5° grid) as input for the crop model also rationalizes the use of SIMPLE over other field scale models. With the increasing size of the area under investigation, input data tend to become more uncertain in relation to the point data from the experimental sites (Parker et al., 2002). Therefore, the model applied should also embrace the uncertainty either by decreasing the sensitivity to highly uncertain inputs or by focusing more on physiological responses that are primarily influenced by available inputs, such as interseasonally or interannually variable climate conditions from SCFs.

To apply the SIMPLE model in our study, we calibrated four cultivar specific parameters among 13 parameters, which consist of nine crop (species)-specific and four cultivar-specific parameters. Calibration was done using the province-level, triennium average yield data ending 2016–2017 from the study by Mottaleb et al. (2018): 3.207 ton/ha in Rangpur, 2.625 ton/ha in Mymensingh, 2.253 ton/ha in Sylhet, 3.117 ton/ha in Rajshahi, 2.931 ton/ha in Khulna, 3.002 ton/ha in Dhaka, 2.931 ton/ha in Khulna, 2.801 ton/ha in Barisahal, and 2.315 ton/ha in Chattagram. The calibrated SIMPLE model resulted in wheat yield simulations less than 10% deviation from the reported yields for all provinces examined. Same as the wheat blast model, the calibration removed the systematic bias of the ERA5-Land reanalysis products over Bangladesh. The parameters of the calibrated SIMPLE model are shown in Supplementary Table 2.

### Seasonal Climate Forecasts and Downscaling

Since 2007, the APEC Climate Center (APCC) has issued multi-model ensemble (MME) SCFs for the upcoming 3- to 6-month overlapping seasons.^1^ The operational APCC MME SCFs are provided in the form of monthly anomaly compared with the climatological period of 1983–2005. Monthly MME forecast data from eight GCMs, in a 2.5° × 2.5° latitude/longitude grid format, were used in the analysis. The selection of GCMs for this study was based on the availability of the most continuous common datasets for the period of 2014–2018. An equal weighing approach was used to determine the contribution of each model ensemble to the final MMEs. Descriptions of the GCMs used are presented in Table 1.

**TABLE 1:** A description of the individual models used in the APEC Climate Center multi-model ensemble forecasts and their spatial resolution.

Model name	Institution	Model resolution	References
CWB	Central Weather Bureau (Taipei)	T42L18	Liou et al., 1997
HMC	Hydrometeorological Centre of Russia (Russia)	1.125° × 1.40625°	Trosnikov et al., 2005
MSC_CANCM3	Meteorological Service of Canada (Canada)	1.41° × 0.94°	Scinocca et al., 2008
MSC_CANCM4	Meteorological Service of Canada (Canada)	1.41° × 0.94°	Merryfield et al., 2013
NASA	National Aeronautics and Space Administration (United States)	288 × 181	Molod et al., 2012
NCEP	Climate Prediction Center, NCEP/NWS/NOAA (United States)	T62L64	Kanamitsu et al., 2002
PNU	Pusan National University (South Korea)	T42L18	Ahn and Kim, 2013
POAMA	Centre for Australian Weather and Climate Research/Bureau of Meteorology (Australia)	T47L17	Lim et al., 2012

In order to use the APCC MME SCFs as input variable in the wheat blast and SIMPLE models, downscaled hourly and daily weather realizations were needed, respectively. For downscaling, the monthly forecasts from individual GCMs were first bias-corrected using the same climatological period (1983–2005) of ERA5-Land reanalysis data using a mean bias correction (MBC) method. MBC searches for the mean error in the model forecasts by comparing with corresponding observation data and adding (or subtracting) it from each variable dataset to have zero mean error (Marcos et al., 2018). MBC also leads to spatial downscaling of the original model forecasts from GCMs (2.5° × 2.5°) into the high resolution of the ERA5-Land (0.1° × 0.1°), which enabled more location-specific application of SCFs in our study. Using the bias-corrected forecasts’ monthly means of temperature and precipitation variables, we subsequently selected a best-fit historical observation data, in this case from the ERA5-Land reanalysis data for 30 years of 1981–2010, based on the Mahalanobis Distance (MD) (Cho et al., 2016). Similar analog sampling from historical observation data has been frequently used for the downscaling of GCM data for subsequent applications (Hirschi et al., 2012; Wu et al., 2012). The best-fit observation data having minimum MD score and its corresponding hourly and daily data are retrieved and used to simulate the wheat blast and SIMPLE models, respectively.

### Wheat Blast Risk and Wheat Yield Simulations

The calibrated wheat blast model was used to examine whether the model can reproduce the disease outbreaks during the seasons of 2015–2016 and 2016–2017. Hourly weather variables (temperature, relative humidity, and rainfall) from the ERA5-Land reanalysis data were extracted for individual grids and used as input variables of the wheat blast model. The resulting grid-based wheat blast risks were aggregated to the district boundaries of Bangladesh (DIVA-GIS^2^). Since the wheat blast model runs for 60 days from planting date, we determined the planting date for winter wheat in Bangladesh based on crop calendars reported on the literature and the FAO GIEWS Country Brief on Bangladesh (FAO, 2008; Hossain et al., 2013; Bangladesh Bureau of Statistics [BBS], 2018). As a result, we set the 10th of December as a fixed planting date of winter wheat in all districts in Bangladesh.

Since the calibrated SIMPLE model requires daily scale weather variables of maximum/minimum temperature, rainfall amount, and solar radiation, we produced them by aggregating the hourly ERA5-Land reanalysis data. Although the observed wheat yields to be compared with the model simulations are based on the unknown actual distribution of planting dates, we assumed the actual planting dates to be around the 10th of December for the purpose of model simulation in the study. In this regard, we used the 10th of December as a fixed planting date. District-specific soil characteristics, such as fraction of plant available water-holding capacity in considered soil bucket (AWC), runoff curve number (RCN), deep drainage coefficients (DDC), and active main root zone depth (RZD), were extracted from the Harmonized World Soil Database v 1.2 at 0.05° spatial resolution (Wieder et al., 2014) and used to run the SIMPLE model.

### What-if Scenario Analysis Using the SCF-Based Early Warning Service for Wheat Blast

Evaluating what-if scenarios for possible seasonal disease prediction-based interventions, we examined alternative planting dates to avoid the high-risk periods of wheat blast while maintaining comparable wheat yield level to the one with normal planting date in the 2016–2017 seasons. This exercise was to find optimal trade-off points between reducing wheat blast risk and securing wheat yield. Because there is no modeling linkage made between two models, we had to simulate both models separately. Downscaled SCFs from eight GCMs were used to run the wheat blast and SIMPLE models with a range of planting dates (dekadal interval) within the planting window of Bangladesh, which is from November to early January (FAO, 2008). To secure a sufficient lead time, we used 6-month SCFs for November–April, which is issued by the end of October. Eight simulated wheat blast risk scores and yield predictions from eight GCM SCFs were visualized as a box plot to compare with observed ones from the ERA5-Land reanalysis data, respectively. For the what-if scenario analysis, potential yield loss impacts were estimated based on the reported relationships between the wheat blast intensity and the corresponding yield loss from literature review.

### Predictability and Potential Value of the SCF-Based Early Warning Service for Wheat Blast

The predictability of the SCF-based early warning service was evaluated by comparing the observation (the ERA5-Land)-based and the SCF-based wheat blast risk scores for 1983–2005 (the common hindcast period of eight GCM SCFs). In addition, the 3-year average observation-based wheat blast risk scores for the period from t–3 to t–1 were produced, assuming that it represents the anticipated wheat blast risk for the coming season. It works as the reference when measuring the added values of the SCF-based early warning service that considers additional sources of information, which is the SCFs. We utilized the Pearson’s temporal correlation coefficient (TCC) and RMSE to compare the predictabilities of the SCF-based wheat blast risk scores and the reference risk scores.

We also examined the potential value of the SCF-based early warning service for wheat blast in Bangladesh by simulating a rule-based approach toward predicted risks from the early warning service. In this study, we define that the risk-averse decision changes planting dates based on the SCF-based wheat blast risk and yield simulations to minimize the predicted risks, whereas the risk-disregarding decision refuses to change but sticks to the conventional calendar-based planting date irrespective of the early warning. The examination was done for 1983–2005, during which we run the wheat blast and SIMPLE models with a range of planting dates (dekadal interval) from November to early January. Our rule for planting date selection is similar to the previous what-if scenario analysis. We chose a planting date with the lowest wheat blast risk prediction, but having a comparable yield, more than 90% of the predicted yield from the normal planting date (the 10th of December). Subsequently, we compared the actual yields with the changed planting date and with the normal planting date, which are simulated using the ERA5-Land observation data, to evaluate the long-term benefit of the SCF-based early warning service. In this way, we directly compared the risk-averse decision (changing planting date) with the risk-disregarding decision (clinging to the normal planting date irrespective of the SCF-based early warning). Based on the previous studies (Islam et al., 2016; Mottaleb et al., 2018), we defined the artificial crop loss ratio to each unit of wheat blast risk score. Considering the reported severity level and wheat growing areas per district (Islam et al., 2016) and the reported total yield loss due to wheat blast outbreaks in the 2015–16 seasons (Mottaleb et al., 2018), it was derived that one unit (1) of risk score corresponds to 10.88% yield loss. This artificial crop loss ratio was used to calculate the estimated actual crop yield (attainable yield - crop loss due to wheat blast) for both risk-averse and risk-disregarding decisions. The yield loss was calculated separately for each province as the wheat growing area per province played a role of weighing factor when converting the risk score to the yield loss.

## Results

### Reproducing Wheat Blast Outbreaks Over Bangladesh Using the Wheat Blast Model

**FIGURE 1:**
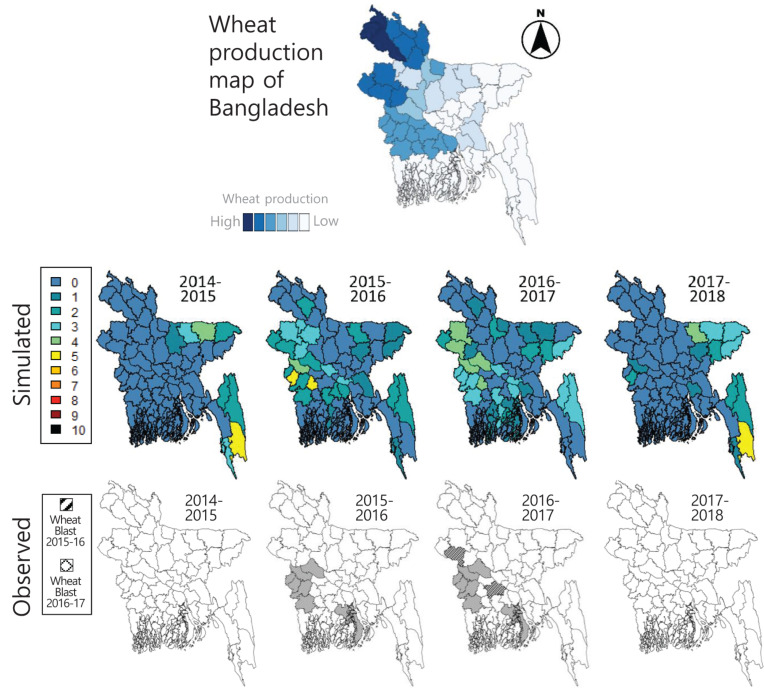
Simulated wheat blast risk probabilities in the districts of Bangladesh from the 2014–15 seasons to the 2017–18 seasons using the calibrated wheat blast model developed by Fernandes et al. (2017) and calibrated in the study. Geographical distribution changes of the wheat blast outbreaks in the districts of Bangladesh during the same period are also shown for comparison with the simulated results. On the top box, wheat production map of Bangladesh shows the major wheat-producing districts, adapted from Sadat and Choi (2017), to be compared with the simulated and observed wheat blast maps.

The calibrated wheat blast model successfully simulated the spatial pattern of disease risk probabilities in the districts of Bangladesh from the 2014–2015 to the 2017–2018 wheat growing seasons (Figure 1). A resulting RMSE between the normalized disease risk simulations and the normalized observed disease intensities of the 2015–2016 seasons for all eight districts was 0.15, with 0.05 of minimum absolute error and 0.37 of maximum absolute error. Graphical comparison with the observed distribution of wheat blast outbreaks across Bangladesh revealed that the calibrated wheat blast model has reasonably good performance (Figure 1). Relatively higher risk probabilities were simulated on the most affected districts in the 2015–2016 and 2016–2017 seasons, whereas lower risk probabilities were simulated in other unaffected districts. In the 2014–2015 and 2017–2018 seasons when there were no (or negligible) reported wheat blast cases, the model simulated very low risk probabilities not only on the affected 10 districts but also on other districts in Bangladesh, except for multiple east districts consistently resulting in some level of unrealized risk probabilities, primarily due to the absence of wheat blast fungal inoculum.

### Predictability of the SCF-Based Early Warning Service for Wheat Blast

**FIGURE 2:**
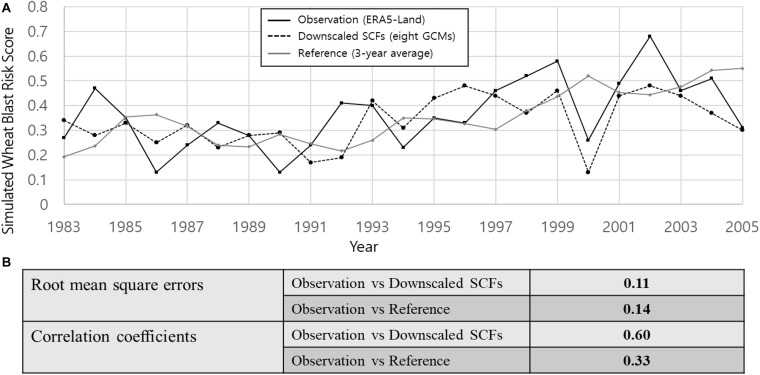
Comparison of the simulated wheat blast risk scores from the observation (ERA5-Land), the downscaled SCFs from eight GCMs, and the reference (3-year average) for the period of 1983–2005. **(A)** Graphical comparison of the time series wheat blast risk scores produced by the wheat blast model using all three input data. **(B)** Note the root-mean square errors and correlation coefficients between the observation and downscaled SCFs and between the observation and reference.

Figure 2 shows the graphical comparison between the wheat blast model simulations using the downscaled SCFs, the reference, and the ERA5-Land data for the period of 1983–2005. The 3-year average observation-based wheat blast risk scores as the reference were presented to measure the added values (i.e., predictability) of the SCF-based early warning service in the study. Overall, the simulated wheat blast risks gradually increased during the period. The time series graphs demonstrated that the SCF-based disease risk simulations showed better temporal correlations with the ERA5-Land data, indicating relatively higher predictive skills for the wheat blast risks (Figure 2A). Statistical analyses resulted in smaller RMSE and larger TCC between the results from the ERA5-Land and the downscaled SCFs, in comparison to the ones from the ERA5-Land and the reference (Figure 2B). This may indicate that the interannual variability of the wheat blast risks depended strongly on the climate condition in December–February, and the relatively good predictability of the climate variables in the SCFs resulted in the potential predictability. The comparison between the results from the SCFs and those of the reference also indicated that the prediction using the SCFs may have some benefits over using the simple reference method.

### What-if Scenario Analysis With the SCF-Based Early Warning Service for the 2016–2017 Wheat Blast Outbreaks in Bangladesh

**FIGURE 3:**
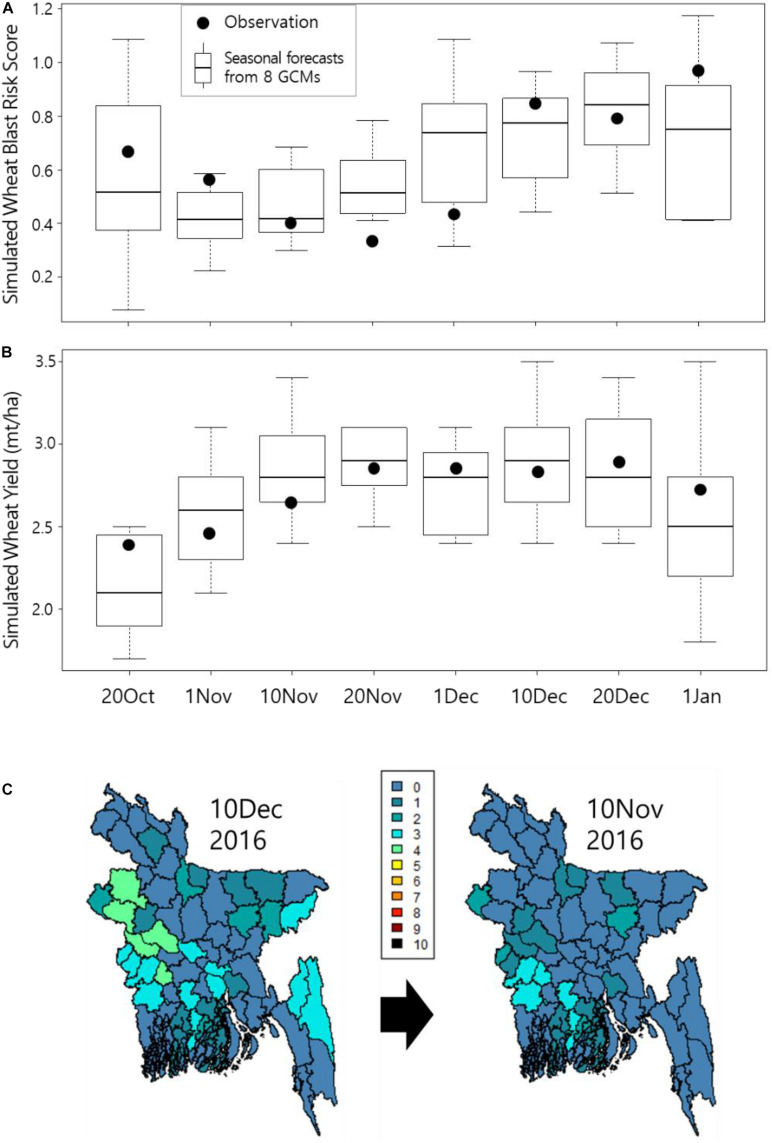
Comparison of simulated wheat blast risk scores **(A)** and wheat yields **(B)** using both the downscaled SCFs (boxplots) and the observation from the ERA5-Land reanalysis data (black dots) over a range of different planting dates with a dekadal interval from October 20, 2016 to January 1, 2017. Note that wheat blast risk scores were significantly reduced when the planting date was changed from December 10 to November 10 **(C)**.

Here, the what-if scenario analysis applies for the 2016–2017 wheat growing seasons, where we reasonably assume that policy-makers and farmers are already aware of the existing wheat blast risk from the 2015–2016 outbreaks in the affected districts, and there is a SCF-based early warning service for wheat blast that aids the decision-making for the selection of planting dates. The SCF-based early warning service indicated that planting wheat on the 10th of November, 2016 resulted in the lowest wheat blast risk score compared with other planting dates and the normal planting date of the 10th of December, 2016 (Figure 3A), yet maintaining more than 90% of yield compared with the normal planting date (Figure 3B). Subsequent simulations using the ERA5-Land observation data also confirmed that the planting on the 10th of November resulted in less risk score over the affected districts than that on the 10th of December planting, yet maintaining minimum yield loss (about 0.15 ton/ha less yield simulated). It was noted that the wheat blast risk scores from the observation showed greater variability than those from the eight SCF-based simulations, whereas both wheat yield simulations showed similar variability over the examined planting dates except for the 20th of October. Yield decline during early planting dates is primarily caused by the marginal weather condition affecting crop performance due to early planting (Figure 3B). Graphical comparison between the two planting dates was made, and it clearly confirmed the significantly reduced wheat blast risks in most districts with the changed planting date of the 10th of November (Figure 3C). Overall, the SCF-based simulations for wheat blast risk and yield showed similar variations to the observation data-based simulations, indicating relatively good performance of the SCF-based early warning. However, different from what was expected from the SCF-based predictions, planting on the 20th of November seemed to result in the lowest wheat blast risk score with one of the highest wheat yields among all planting dates. This indicates the inevitable uncertainty in the SCF-based simulations.

Estimating from the previous studies, in which an average yield loss of 24.5% in the affected 15,000 ha wheat growing areas in eight districts was reported (Islam et al., 2016; Mottaleb et al., 2018), we interpolated the possible yield loss in the 2016–2017 seasons upon the relative risk score differences between the 2015–2016 and the 2016–2017 seasons, assuming that the simulated risk score has a significant correlation with the resulting yield loss. This approach has no choice but to be theoretical, as it assumes many critical factors due to limited data available. Our results showed that (1) without proactive intervention aided by the SCF-based early warning service for wheat blast (with planting on the 10th of December), about 31.1% of an average yield loss was estimated in the 2016–2017 seasons, whereas (2) with proactive intervention (changing planting date to the 10th of November), about 15.7% of an average yield loss was estimated. Combining the yield loss generated due to the marginal planting on the 10th of November, our final estimation indicates that the changing planting date based on the SCF-based early warning service could have avoided about 11% yield loss out of the 31.1% in the 2016–2017 seasons due to potential wheat blast outbreaks.

### Potential Value of the SCF-Based Early Warning Service for Wheat Blast

In the study, the potential value of the risk-averse decision based on the SCF-based early warning service over the risk-disregarding decision was evaluated (Table 2). Our rule for planting date selection by the risk-averse decision led to selecting the planting date with the lowest wheat blast risk but not sacrificing attainable yield that was predicted using the SCFs, whereas the risk-disregarding decision planted only on the normal planting date (the 10th of December) irrespective of the SCF-based early warning information. In fact, the observed results in Table 2 showed the positive outcome of the applied rule, where the risk-averse decision resulted in less wheat blast risk score and thus less yield loss, while maintaining comparable attainable yield to the risk-disregarding decision (2.97 vs 3.05 ton/ha). In other words, many of the expected predictions in the wheat blast risk and attainable yield based on the SCF-based early warning service ended up being realized in the observations. This is very promising findings when it comes to the predictability of both SCF applied and the resulting model outputs. More importantly, the long-term average of actual yields (attainable yield - yield loss due to wheat blast) was higher in the risk-averse decision than in the risk-disregarding decision, where more years showed positive outcome for the former (14 out of 23 years). This result surely encourages the users to keep placing their trust on the information from the SCF-based early warning service and thus makes sure that the service is sustainable and increases the adoption rate by more users at the end.

**TABLE 2:** Potential value of the risk-averse decision over the risk-disregarding decision for the SCF-based early warning service for wheat blast, based on simulations for the period of 1983–2005.

	Planting date(s)	Wheat blast risk score	Yield loss (%)	Attainable yield (ton/ha)	Actual yield (ton/ha)	Number of years with better actual yield
Risk-averse decision	Selected date between November 10 and January 1	0.13	1.51	2.97	2.93	14/23 (61%)
Risk-disregarding decision	December 10	0.45	5.24	3.05	2.89	9/23 (39%)

## Discussion

### Potential Applicability and Benefit of the SCF-Based Early Warning Service for Wheat Blast

This retrospective study showed the feasibility of the SCF-based early warning service for the pre-season strategic interventions of forecasted crop diseases. Applicability and benefits are mostly governed by the forecast accuracy and lead time for advanced decision-making. Our results indicate that the SCF-based early warning service has both predictability and added value over conventional decision-making process not considering forecast information as demonstrated with the 3-year average reference (Figure 2). This is partly because the models used in the study were fairly sensitive enough to translate the variable climate conditions from the SCFs into respective disease risk and yield information. Another possibility is in using the MME, as averages of ensemble forecasts are considered more skillful than single-model forecast averages because multiple models can average out errors of individual models (Stockdale et al., 2010; DelSole and Tippett, 2014).

The models used in the study require different temporal resolution weather data (hourly data for the wheat blast model and daily data for the SIMPLE crop model). This may explain the larger variability observed in the wheat blast risk scores than in the wheat yield simulations over the examined planting dates (Figures 3A,B). Sparks et al. (2011) also showed larger variabilities in the metamodels using higher temporal resolution weather inputs. The difference in variabilities between the wheat blast risk scores from both the observation and the SCFs indicates the importance of temporal downscaling of the low-resolution SCFs in the SCF-based early warning service. Another way to overcome the temporal discrepancy is a metamodeling approach by adapting models to use lower resolution input data (Sparks et al., 2011). Either downscaling or metamodeling will result in added uncertainty in the early warning information; therefore, it is important to properly present to users not only the averaged warning information but also the uncertainty in a quantitative way.

Predicted risks for the wheat blast disease from the SCF-based early warning service can potentially be used to enable decision makers to develop informed farm management strategies. Not only such strategic decisions include short-to-mid-term ones (e.g., the selection of resistant cultivars against the high-risk predictions, the seasonal scheduling of chemical and cultural control methods, or identifying the ideal time to plant crops to minimize crop losses due to diseases) but also decisions that do not directly pertain to disease management but have many crop health consequences (e.g., choices involved in the type of crop establishment, crop rotation, or cropping systems). Nevertheless, few studies demonstrated the clear applicability of the SCF-based early warning services (Meinke and Stone, 2005; Choi et al., 2015), primarily due to large uncertainties that not only the GCM forecasts and applied agro-models but also the input variables and operational processes, such as downscaling or aggregation, fundamentally have. Indeed, users often remain skeptical of the SCF-based information because of individual negative experiences in the pasts (Changnon et al., 1988; Hu et al., 2006). Although our study did not dissect the uncertainties from individual sources, we successfully showed the promising potentials of utilizing SCF-based early warning service in the long-term as demonstrated for the period of 1983–2005 (Table 2), which clearly showed the users with short-term negative experiences that the long-term risk-averse approach will lead to long-term benefit at the end. The rationale for this approach is that consistency eventually pays off in the long run (Crane et al., 2010). Informed decision-making based on the reliable early warning service is essential to cope with future wheat blast epidemics in Bangladesh, which will possibly re-occur in the coming years when favorable environment and susceptible wheat cultivar are present again.

### Limitations of the SCF-Based Early Warning Service for Wheat Blast

There are some remaining areas to be re-visited for improvement. Especially, securing more ground truth data for the disease incidences with metadata, such as location and time, and critical and dynamic factors consisting of abiotic and biotic stressors and agronomic practices affecting the actual yield is among the most urgent follow-ups. For example, the total wheat growing areas in the eight districts were significantly decreased in the 2016–2017 growing seasons due to government guidance to avoiding wheat and changing to other alternative crops (Islam et al., 2019), based on which our resulting figures should be adjusted. Long-term ground truth wheat blast and yield data per district are needed to improve the accuracy of the wheat blast and SIMPLE models. Extensive data also enable the development of crop-disease-coupled models for more realistic simulations based on the given SCFs. In this study, the model calibration was done only using 1-year data that are reported on the literature, resulting in the lack of robustness of the parameters of each model. To increase the validity and applicability of the models, more ground truth data should be used for the model refinement, which will eventually reduce the uncertainty derived from the model performance.

Although the SIMPLE crop model is both applicable to data-scarce areas and realistic with comparable performance to several wheat models (Asseng et al., 2015; Zhao et al., 2019), there are a number of limitations that should be addressed when it happens to simulate more complex dynamics of agroecosystems. The lack of soil–crop nutrient function will potentially cause erroneous simulations for many low input systems that are common in developing countries. Some effects from agronomic practices are not included, such as planting space and depth, which could have some impact on crop yield. More relevant to our study is the lack of pest and disease damage functions in the SIMPLE model. While follow-up studies should take advantage of the simple and easily extendable features of the SIMPLE model by adding pest and disease damage functions and any necessary physiological response functions to overcome its limitations, readers need to be aware of these limitations when interpreting the results in this study.

The value of the SCF-based early warning service depends on a wide range of complex and interrelated factors. These include SCF accuracy, e.g., accuracy at relevant spatial resolutions and lead times, forecast adoption rates and the attitudes of farmers to coping risks, and the actual seasonal conditions that are experienced. Therefore, no matter how skilled a climate forecast is, it is not possible for farmers to eliminate all impacts of climate on production, and any actions that they take to mitigate the risks will cost money, as will any decisions they make based on incorrect forecasts. It is unlikely that SCFs will ever achieve complete certainty in forecasting because of the many chaotic and non-deterministic features of climate systems. In fact, due to the anxiety of users regarding the predictability, many systems that have been developed still only use yield estimates from past periods, such as the 3-year average reference in our study, rather than using actual yield estimates to support informed decision-making.

### Potential Ways Forward

Through the advances in GCM modeling, state-of-the-art downscaling techniques, and the wide application of big-data analyses and statistical techniques, such as the Bayesian-based parameter estimation in the climate and agricultural research fields (Gronewold et al., 2009; Jeong et al., 2016), it is expected that the use of SCF and agricultural modeling in agricultural decision-making will increase in the future. In addition, recent increases in the availability of crop/pest/disease-related data at national to global level would help improve the SCF-based early warning services. Such global datasets include the gridded historical yield time series (Iizumi and Sakai, 2020), global-scale, quantitative, standardized information on crop losses (Savary et al., 2019), potential sowing and harvesting windows (Iizumi et al., 2019), and high-resolution crop phenology (Luo et al., 2020). To develop comprehensive developmental frameworks for the SCF-based early warning services, it is also necessary to consider and simultaneously achieve all three of the key prerequisites for climate services: credibility, salience, and legitimacy (Cash et al., 2003).

## Data Availability Statement

Publicly available datasets were analyzed in this study. This data can be found here: APEC Climate Center ADSS.

## Author Contributions

K-HK designed and conducted the experiments, and wrote the manuscript. EDC conducted literature review and data clean-up and analysis.

## Conflict of Interest

The authors declare that the research was conducted in the absence of any commercial or financial relationships that could be construed as a potential conflict of interest.

## Footnotes

1 www.apcc21.org

2 http://www.diva-gis.org/gdata
