# Supplementary material for: Retrospective Study on the Seasonal Forecast-Based Disease Intervention of the Wheat Blast Outbreaks in Bangladesh
Source: Front Plant Sci. 2020 Nov 23;11:570381. doi: 10.3389/fpls.2020.570381 (PMC7719836; doi:10.3389/fpls.2020.570381)
Supplement: Supplementary file 1 [file Table_1.DOCX]

**Supplementary Table S1.** Calibrated parameters of the wheat blast model compared to the original parameters in Fernandes et al. (2017).

| Model Function | Parameter | Original Parameter | Calibrated Parameter |
| --- | --- | --- | --- |
| Inoculum Potential (IP)  ◌ 14.35 - 0.25 * T,  if 15˚C < T < 27˚C and RH > 93%;  ◌ -8.5 + 0.59 * T,  if 27˚C < T < 35˚C and RH > 93%;  ◌ 0, otherwise. | *Low T (˚C)* | 15 | 16 |
|  | *Medium T (˚C)* | 27 | 29 |
|  | *High T (˚C)* | 35 | 37 |
|  | *RH* *(%)* | 93 | 90 |
| Day Favoring Infection (DFI):  ◌ Daily max T > 23 ˚C,  amplitude > 13 ˚C, and  daily mean RH > 70%. | *Daily max T (˚C)* | 23 | 25 |
|  | *Amplitude (˚C)* | 13 | 10 |
|  | *Daily mean RH (%)* | 70 | 65 |
