# Supplementary material for: Retrospective Study on the Seasonal Forecast-Based Disease Intervention of the Wheat Blast Outbreaks in Bangladesh
Source: Front Plant Sci. 2020 Nov 23;11:570381. doi: 10.3389/fpls.2020.570381 (PMC7719836; doi:10.3389/fpls.2020.570381)
Supplement: Supplementary file 2 [file Table_2.DOCX]

**Supplementary Table S2.** Calibrated parameters of the SIMPLE crop model compared to the original parameters in Zhao et al. (2019).

| Parameter | Original Parameter | Calibrated Parameter |
| --- | --- | --- |
| *T_sum_* | 2200 | 2350 |
| *HI* | 0.36 | 0.27 |
| *I_50A_* | 480 | 410 |
| *I_50B_* | 200 | 120 |

- *Tsum*: Cumulative temperature requirement from sowing to maturity (˚C d).

- *HI*: Potential harvest index.

- *I_50A_*: Cumulative temperature requirement for leaf area development to intercept 50% of radiation (˚C d).

- *I_50B_*: Cumulative temperature till maturity to reach 50% radiation interception due to leaf senescence (˚C d)
